# The 0-3 Lead Zirconate-Titanate (PZT)/Polyvinyl-Butyral (PVB) Composite for Tactile Sensing

**DOI:** 10.3390/s23031649

**Published:** 2023-02-02

**Authors:** Eun-Bee Jo, Yoon-A Lee, Yoon-A Cho, Paul A. Günther, Sylvia E. Gebhardt, Holger Neubert, Hyun-Seok Kim

**Affiliations:** 1 Division of Electronics and Electrical Engineering, Dongguk University-Seoul, Seoul 04620, Republic of Korea; 2 Department Smart Materials and Systems, Fraunhofer IKTS, Fraunhofer Institute for Ceramic Technologies and Systems, 01277 Dresden, Germany

**Keywords:** tactile sensor, screen printing, lead zirconate-titanate (PZT), polyvinyl-butyral (PVB), 0-3 composite

## Abstract

In this study, a 0-3 piezoelectric composite based on lead zirconate-titanate (PZT)/polyvinyl-butyral (PVB) was fabricated and characterized for its potential application in tactile sensing. The 0-3 composite was developed to incorporate the advantages of both ceramic and polymer. The paste of 0-3 PZT–PVB composite was printed using a conventional screen-printing technique on alumina and mylar substrates. The thickness of the prepared composite was approximately 80 μm. After printing the top electrode of the silver paste, 10 kV/mm of DC field was applied at 25 °C, 120 °C, and 150 °C for 10 min to align the electric dipoles in the composite. The piezoelectric charge coefficient of $d_{33}$ and the piezoelectric voltage coefficient of $g_{33}$ were improved by increasing the temperature of the poling process. The maximum values of $d_{33}$ and $g_{33}$ were 14.3 pC/N and 44.2 mV·m/N, respectively, at 150 °C. The sensor’s sensitivity to the impact force was measured by a ball drop test. The sensors showed a linear behavior in the output voltage with increasing impact force. The sensitivity of the sensor on the alumina and mylar substrates was 1.368 V/N and 0.815 V/N, respectively. The rising time of the sensor to the finger touch was 43 ms on the alumina substrate and 35 ms on the mylar substrate. Consequently, the high sensitivity and fast response time of the sensor make the 0-3 PZT–PVB composite a good candidate for tactile sensors.

## 1. Introduction

Tactile sensing is an essential tool used in instrumentation electronics for evaluating physical parameters such as stress, strain, pressure, and force [1,2,3]. A wide range of technologies, including resistive, capacitive, piezoelectric, triboelectric, optical, and magnetic sensors are available for tactile sensing. Among them, piezoelectric sensors have received significant attention due to their low power consumption compared with other types of sensors [4].

Piezoelectric materials cause electrical polarization when stress is applied or mechanical deformation when an electric field is applied. Thus, in piezoelectric materials, mechanical energy can be converted into electrical energy and vice versa. Piezoelectric ceramics including barium titanate (BaTiO_3_), lead titanate (PbTiO_3_), and lead zirconate-titanate (PbZr_x_Ti_1−x_O_3_, 0 < x < 1, PZT) are often used in the fabrication of piezoelectric sensors because of their high piezoelectric charge coefficient ($d_{33}$). In general, PZT has $d_{33}$ > 300 pC/N and BaTiO_3_ has $d_{33}$ > 200 pC/N, depending on the process and synthesis conditions [5,6,7].

However, piezoelectric ceramics are difficult to use in a variety of environments owing to their restricted formability, hardness, and brittleness [8]. Studies on piezoelectric composites made of piezoelectric ceramic and polymer have been actively conducted to improve and compensate for these limitations [1,9,10,11,12,13,14,15,16]. These diphasic composites were developed to combine the high piezoelectric charge coefficient and dielectric constant of piezoelectric ceramics with the flexibility of the polymers. According to Newnham et al. [11], piezoelectric ceramic–polymer composites can be classified into 10 categories (0-0, 0-1, 0-2, 0-3, 1-1, 1-3, 2-1, 2-2, 2-3, and 3-3) depending on the type of connectivity in the active phase (ceramic material) and matrix phase (polymer). The process and synthesis conditions of the composites vary based on the type of connectivity. The simplest composites so far studied are those with 0-3 connectivity, which were first developed by Kitayama and Sugawara using PZT powder and polyvinylidene fluoride [9]. In this study, 0-3 connectivity, which consists of ceramic particles (active phase) randomly dispersed in the polymer (matrix phase), was chosen due to its simple fabrication process and the fact that functional properties can be easily modulated by varying the filler content [10]. 

PZT is widely used for the active phase in 0-3 piezoelectric composites owing to its low cost and high piezoelectric coefficient, and several different polymers have been investigated in combination with PZT in previous studies. These polymers include polyvinyl-butyral, polydimethylsiloxane, epoxy, polyamide, and polyurethane [17,18,19,20,21]. Polyvinyl-butyral (PVB) is a colorless, amorphous thermoplastic resin, which is often utilized in technological applications such as paints and pastes. Because of its excellent flexibility, good adhesion property, workability, and easy production, PVB is an excellent candidate for use in composites [22]. In addition, the ferro/piezoelectric characteristics of PZT can be tuned towards hard or soft characteristics by means of proper doping [23,24]. The terms “soft” and “hard” PZT ceramics refer to the mobility of the dipoles or domains, as well as to the polarization and depolarization behaviors. The advantages of the hard PZT materials are their large piezoelectric coupling factors, high mechanical qualities, and stability to high electrical stress which make them interesting for high-power acoustic applications. Whereas soft types are suitable for actuators and sensors with high sensitivity because they can be polarized easily even at relatively low electric field strengths and have large piezoelectric coefficients. Based on these properties, modified PZT of the soft type was selected to fabricate a tactile sensor with great sensitivity.

Studies on 0-3 composites made of PZT and PVB focused only on piezoelectric and dielectric properties but not on their applications as sensors [25,26]. A technical study was conducted about screen-printed 0-3 piezoelectric composites on different substrates (polymers, printed circuit boards, and fabrics) [27]; however, there are only limited studies on the influence of substrate material on functional properties for tactile applications. As a sensor component, a substrate will affect the sensor’s sensitivity and thus must be considered by performance evaluation for tactile sensing [28].

In this study, we fabricated sensors with 0-3 PZT-PVB composites on two different types of substrates via the screen-printing technique. The influence of poling temperature (25 °C, 120 °C, and 150 °C) on piezoelectric characteristics was examined using the quasi-static method. The relative permittivity of the composites was determined through impedance measurement to calculate piezoelectric voltage coefficient. The maximum voltage across the composites was measured during a ball drop test to examine the sensor’s response to the impact force and the linear regression model was used to calculate the sensitivity of the sensor. Finger touch was used to evaluate the sensor’s tactile sensing capacity. Eventually, the application of the 0-3 PZT–PVB composite as a tactile sensor was demonstrated.

## 2. Materials and Methods

### 2.1. Materials

**Figure 1:**
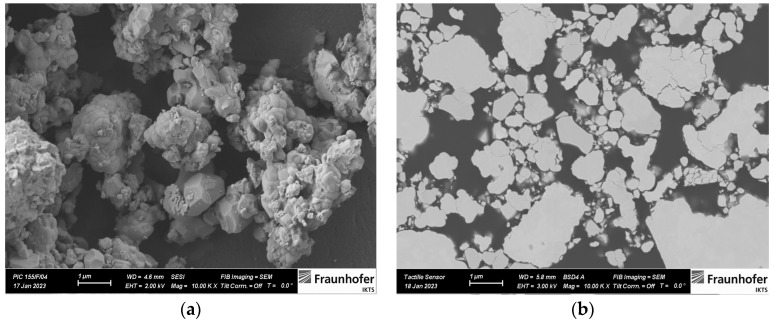
Field emission scanning electron microscopy images: (**a**) PZT particles; (**b**) 0-3 PZT–PVB composite.

PZT ceramic powder (PIC 155) was provided by PI Ceramics GmbH, Germany. PVB was purchased from TER HELL & Co. GmbH, Germany. Mylar substrate (foil siliconized on the backside) was provided by Laufenberg GmbH, Germany. Alumina substrate (Rubalit 710, 99.6%) was purchased from CeramTec GmbH, Germany. All materials were used as received without further purification. The PZT powder properties can be found in the corresponding data sheet [29]. The morphology of the PZT particles was observed with a field emission scanning electron microscope (FESEM) as shown in Figure 1a. Based on the images, the diameter of the PZT ceramic powder was in the range of 1–2 µm and the size of agglomerated particles was below 10 µm.

### 2.2. Fabrication of Tactile Sensor

A tactile sensor was fabricated in a sandwich structure with silver electrodes at the top and bottom of the composite. The PZT powder was uniformly dispersed within a PVB-based binder solution using a three-roll mill to fabricate the 0-3 PZT–PVB composite. The PZT powder of the paste was 80% by mass. The agglomerated particle sizes of less than 5 μm in the paste were measured with a grindometer. This was further confirmed by the backscattered FESEM of piezocomposite (Figure 1b). Ag-based paste with 85% Ag by mass was used for electrodes.

**Figure 2:**
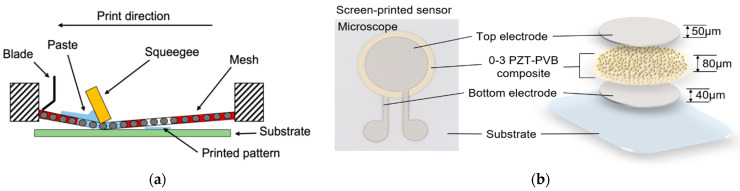
(**a**) Schematic of the screen-printing process; (**b**) microscope image of the screen-printed sensor (left) and schematic of sensor’s structure (right).

The layout of the tactile sensor was designed using CAD software (DraftSight). A set-up of bottom electrode, piezocomposite, and top electrode was subsequently printed on alumina substrate and on mylar substrate, respectively, using the screen-printing technique. Alumina is a rigid ceramic substrate with a thickness of 0.63 mm, whereas mylar is a transparent, flexible polymer substrate with a thickness of 0.075 mm. To print a pattern using the screen-printing technique, the pastes are placed on the mesh using a blade after an emulsion process, which is the process of creating a pattern in a mesh, and pressure is applied with a squeegee to print the paste on the substrate, as depicted in Figure 2a. This simple method can be used to control the thickness of the film. To achieve the target thickness, the top and bottom electrodes were screen printed once, and the 0-3 PZT–PVB composite was screen-printed three times. The printed pastes on the substrate were leveled and planarized for 5 min at room temperature (25 °C), and then they were dried for 1 h at 150 °C in the oven.

As shown in Figure 2b, the thicknesses of the layers of the screen-printed sensor were as follows: the bottom electrode was ~40 μm, the piezocomposite was ~80 μm, and the top electrode was ~50 μm. After deposition and drying, the mylar substrate and the alumina substrate were cut to a size of 1.8 cm × 2.0 cm to separate each of the sensors for poling and measurement. The diameter of the composite was set 1 mm wider than the diameter of the electrodes (5 mm, 10 mm, and 15 mm) to prevent short circuits between the top and bottom electrodes.

High DC voltage source equipment (HV-Tester UG36, ETL Prüftechnik GmbH, Korntal-Münchingen, Germany) was used to pole the composites. This poling process was carried out in order to align ferroelectric domains in PZT particles in the direction of the electric field and increase the piezoelectricity [30]. The piezocomposites were poled for 10 min in the air at 25 °C, 120 °C, and 150 °C with an applied electric field of 10 kV/mm. It is known that poling of piezoelectric particles in a polymer matrix is difficult due to the misfit in dielectric constants of polymer and ceramic. Moreover, polymers have much lower permittivity than ceramic particles, resulting in decreased poling efficiency. A dielectric breakdown could occur if a too high electric field is continuously applied. The poling temperature was increased to lower the polymer’s resistivity, and the voltage drop across the ceramic particles was increased to increase the poling efficiency [31].

## 3. Results and Discussion

### 3.1. Polarization–Electric Field (P–E) Hysteresis Loop

**Figure 3:**
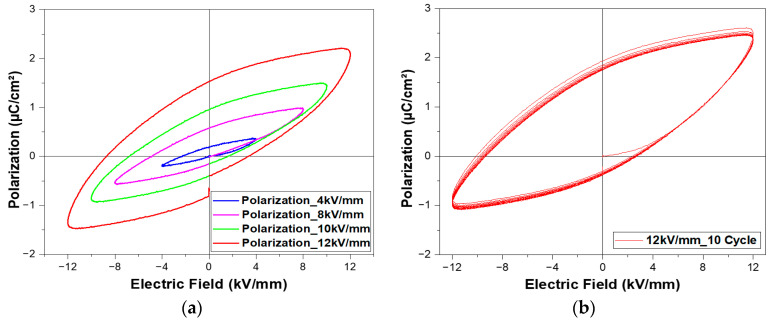
P–E hysteresis loops for 0-3 PZT–PVB composite: (**a**) 4 kV/mm, 8 kV/mm, 10 kV/mm, and 12 kV/mm for 1 cycle; (**b**) 12 kV/mm for a continuous 10 cycles.

Before the poling process, a series of P–E hysteresis loops of the 0-3 PZT–PVB composite was measured at room temperature, indicating the polarization value change under the bipolar electric field. As shown in Figure 3a, different electric fields (4 kV/mm, 8 kV/mm, 10 kV/mm, and 12 kV/mm) were applied to the composite for 1 cycle. The shape of the loops suggests a ferroelectric nature, in which an applied electric field induces rearranged domains in the direction of the electric field [32]. The shape of the loops and their shifts on the P-axis to higher values suggest the presence of electrical conductivities. We determined that the composite reached saturation in an electric field of 12 kV/mm since it was experimentally proven that specimen damage occurred due to dielectric breakdown when the applied electric field exceeded 12 kV/mm. Figure 2b shows the P–E hysteresis for 10 continuous cycles with an applied field of 12 kV/mm. The following saturation properties were obtained: remnant polarization (P_r_) of ~1.94 μC/cm^2^ and coercive field (E_c_) of ~2.72 kV/mm.

### 3.2. Piezoelectric Properties (d_33_, g_33_)

The piezoelectric charge coefficient, d, is one of the parameters for evaluating the piezoelectric material and can be expressed in terms of the charge generated per unit area under an applied mechanical force, as shown in Equation (1):

(1) $$d=\frac{Polarization\;Charge\;Density}{Applied\;Stress}=k\sqrt{\varepsilon_{0}\varepsilon_{r}{}^{T}s^{E}},$$

where k is the electromechanical coupling factor, $\varepsilon_{0}$ is the permittivity of free space $(8.854\times 10^{-12}\;F/m$), $\varepsilon_{r}^{T}$ is the relative permittivity at constant stress, and $s^{E}$ is the compliance under a constant electric field. The piezoelectric charge coefficient $d_{33}$ indicates the polarization generated in the 3-direction when the stress is applied in the 3-direction. In our case, the polarization generated direction and stress applied direction are the same, 3-direction, because the electrodes were at the top and bottom of the composite:

(2) $$d_{33}=\frac{Q}{A}\cdot \frac{A}{F}=\frac{Q}{F}\;\left(C/N\right),$$

where Q is the charge developed by the sensor, A is the conductive area, and *F* is the applied force. The piezoelectric voltage coefficient, $g_{33}$, is also used to assess the ability of piezoelectric materials to generate an electric field per unit of input stress. The $g_{33}$ is related to $d_{33}$ by the following relationship:

(3) $$g_{33}=\frac{Electric\;Field}{Applied\;Stress}=\frac{d_{33}}{\varepsilon_{0}\varepsilon_{r}}\;\left(\mathrm{Vm}/N\right)$$

where $\varepsilon_{r}$ is the relative permittivity. Hence, if the material has a low $\varepsilon_{r}$, a high $g_{33}$ is possible for a given $d_{33}$. $\varepsilon_{r}$ can be determined using the following equation:

(4) $$C=\frac{\varepsilon_{0}\varepsilon_{r}A}{d}\;\left(F\right)$$

where d is the distance between the electrodes, and C is the measured capacitance.

**Figure 4:**
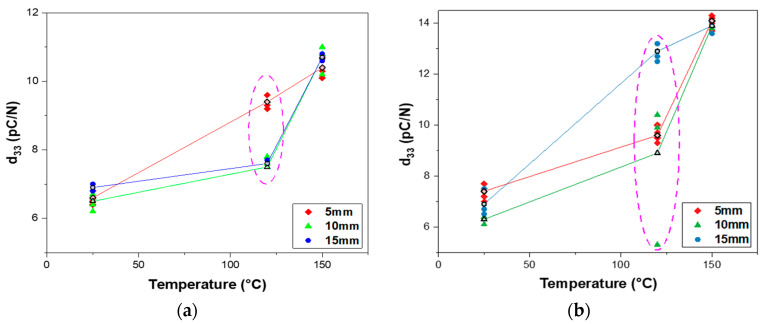
Piezoelectric charge coefficient $d_{33}$ of poled PZT–PVB composites versus poling temperature with various diameters of the electrodes: (**a**) alumina substrate; (**b**) mylar substrate.

After 24 h of aging after the poling process, $d_{33}$ was measured using the Berlincourt meter (ZJ-6B, IACAS, Beijing, China). $\varepsilon_{r}$ was measured using the impedance analyzer (4194A, Keysight Technologies, Inc., Santa Rosa, CA, USA) with a frequency of 1 kHz. The samples on the alumina and mylar substrates were poled at various temperatures to optimize the poling parameters. However, temperatures above 150 °C can cause damage such as wrinkling or bending owing to the limited heat resistance of the mylar substrate. A total of 9 samples for each substrate with different diameters of the electrode (5 mm, 10 mm, and 15 mm) were prepared to confirm the piezoelectric properties. Figure 4 shows $d_{33}$ of the piezocomposites poled at 10 kV/mm for 10 min in the air at different temperatures (25 °C, 120 °C, and 150 °C). On the alumina substrate, $d_{33}$ of 6.7 pC/N at 25 °C, $d_{33}$ of 8.3 pC/N at 120 °C, and $d_{33}$ of 10.6 pC/N at 150 °C were achieved on average (Figure 4a). On the mylar substrate, $d_{33}$ of 6.9 pC/N at 25 °C, $d_{33}$ of 10.5 pC/N at 120 °C, and $d_{33}$ of 14.0 pC/N at 150 °C were obtained on average (Figure 4b). Since $d_{33}$ is a property of the material itself, it is well known that $d_{33}$ is independent of the diameter of the electrodes. However, it was observed that the samples poled at 120 °C had a variation in the $d_{33}$ values when compared with samples poled at other temperatures, as indicated by the circles in Figure 4a,b. This may be a result of aging time, which causes dipoles to naturally align themselves over time compared with the case of right away after poling [32]. However, the reason for this behavior is still under investigation.

Furthermore, we observed that piezocomposites on the mylar substrate had larger and a wider range of $d_{33}$ values than the piezocomposites on the alumina substrate for all poling temperatures. This may be attributed to a bending effect that occurred when samples were fixed by the probes of the $d_{33}$ meter. The rigidity of the alumina substrate prevented any bending effect of samples during the measurement, whereas the flexibility of the mylar substrate resulted in warping of samples under the pressure of the probes during the measurement. However, $d_{33}$ clearly increased with increasing poling temperature. The rate of increase in the $d_{33}$ value was 59.0% for the alumina substrate and 103.8% for the mylar substrate when the composites were poled at 150 °C compared with 25 °C, as shown in Table 1. The maximum values of $d_{33}$ of the composites poled at 150 °C on the alumina and mylar substrates were 11.0 pC/N and 14.3 pC/N, respectively. This result can be explained by the thermally activated transportation mechanism, which increased the composite conductivity and thus the poling efficiency [33].

**Figure 5:**
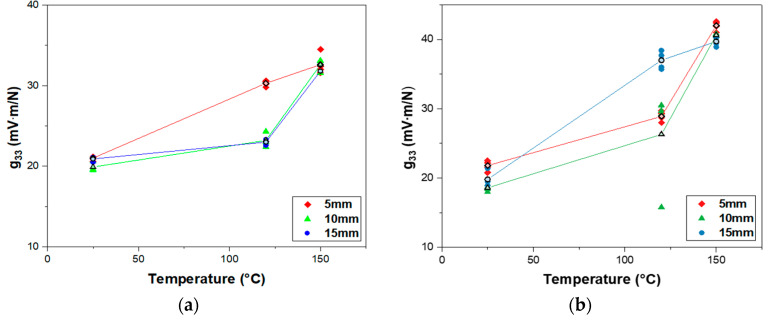
Piezoelectric voltage coefficient $g_{33}$ of poled PZT–PVB composites versus poling temperature with various diameters of the electrodes: (**a**) alumina substrate; (**b**) mylar substrate.

Figure 5 represents $g_{33}$ of the piezocomposites with the same poling conditions as described in Figure 4. $g_{33}$ was calculated with measured $\varepsilon_{r}$ and $d_{33}$ according to Equation (3). The measured $\varepsilon_{r}$ of the poled piezocomposites on the alumina substrate and the mylar substrate was in the range of 34.1 to 38.4 and 37.4 to 40.6, respectively. On the alumina substrate, $g_{33}$ of 20.6 mV·m/N at 25 °C, $g_{33}$ of 25.7 mV·m/N at 120 °C, and $g_{33}$ of 32.4 mV·m/N at 150 °C were obtained on average (Figure 5a). On the mylar substrate, $g_{33}$ of 20.1 mV·m/N at 25 °C, $g_{33}$ of 30.7 mV·m/N at 120 °C, and $g_{33}$ of 40.8 mV·m/N at 150 °C were obtained on average (Figure 5b). The results are in agreement with the data shown in Figure 4 that $g_{33}$ increases with increasing poling temperature, indicating that $g_{33}$ is dependent on $d_{33}$. At the poling temperature of 150 °C, the maximum values of $g_{33}$ for the alumina and mylar substrates were 34.5 mV·m/N and 42.6 mV·m/N, respectively.

**Figure 6:**
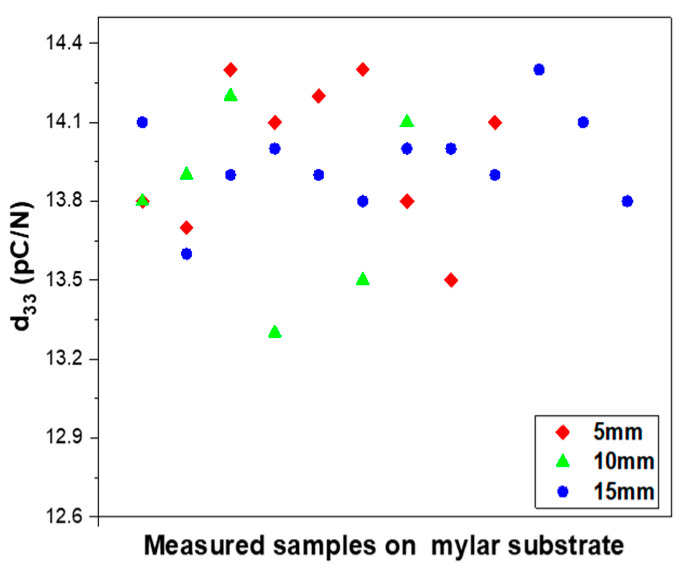
Distribution of the piezoelectric charge coefficient $d_{33}$ of the PZT–PVB composites poled at 150 °C on the mylar substrate with various diameters of the electrodes.

Since the variation in $d_{33}$ values for piezocomposites on the mylar substrate is larger than that on the alumina substrate and because the largest $d_{33}$ was obtained on mylar based samples poled at 150 °C, additional PZT–PVB samples were prepared under the same conditions. Figure 6 shows the distribution of $d_{33}$ values of the PZT–PVB composites poled at 150 °C on the mylar substrate. $d_{33}$ values were between 13.3 and 14.4 pC/N regardless of the diameter of the electrodes, meaning that the piezoelectric property of the composites poled at 150 °C was rather stable.

### 3.3. Impact Force Test

**Figure 7:**
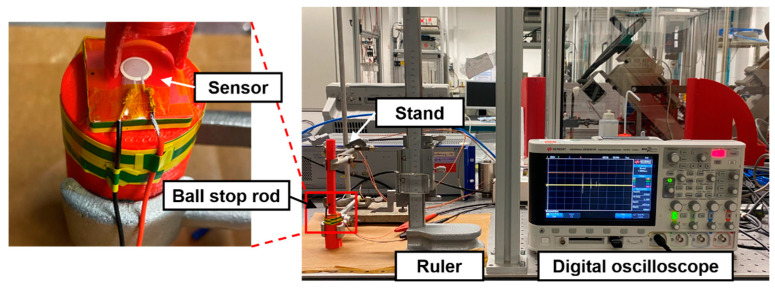
Ball drop test experimental setup for measuring the response of the PZT–PVB composite to impact force.

The ball drop test was used to evaluate our sensor’s responses to the impact force, as shown in Figure 7. The linearity of the response to the increasing force can be simply acquired and compared [34,35,36,37]. The voltage output of the sensor caused by the impact force of the ball was recorded by a digital oscilloscope (DSOX2014A, Keysight Technologies, Inc.). The sensitivity of the sensor to the impact force was also calculated by the ball drop test. A 2.94 g zirconia ball with a diameter of 9.85 mm was dropped on the sensor from various heights of 2 cm, 4 cm, 6 cm, 8 cm, 10 cm, and 12 cm. In ascending order, the free-falling drop of the ball from various heights provided different impact forces. The impact force is typically determined by the mass of the falling object, the surface where the object is dropped, and the impact energy. The mass of the falling ball and the surface of the sensor remained the same in this experiment, but the impact force and velocity increased with cumulative height [37]. The ball was dropped after inserting the ball stop rod into the hole to fit each height of the cylinder tube. The inner diameter of the cylinder tube was 12 mm, and its height was 135 mm. It acted as a fall guide for the ball to have a direct impact on the sensor. The sensor was placed right below the cylinder tube, which was firmly held perpendicularly by the stand [35]. Both the cylinder tube and the specimen support were 3D printed. A ruler was also used to measure the height more precisely. When the ball was dropped from different heights, it exerted a concentrated impact force at the center of the sensor. The impact force produced the compressive stress in the 0-3 PZT–PVB sensing composite, which in turn generated an electric field and output voltage along the vertical direction. The law of conservation of energy was used in the ball drop test to estimate the impact force that would result from a ball drop as follows:

(5) $$\frac{1}{2}mv_{impact}^{2}-\frac{1}{2}mv_{initial}^{2}=mgh$$

where m is the mass of the ball, g is the acceleration of gravity, h represents the height from where the ball is being dropped, and v is the velocity of the ball. The initial velocity ($v_{initial}$) is zero in the falling ball, so the velocity of impact ($v_{impact}$) can be expressed by Equation (6).

(6) $$v_{impact}=\sqrt{2gh}$$

The magnitude of impact force ($F_{impact}$) can be expressed by Equation (7) by substituting $v_{impact}$ into Newton’s Second Law, Ft=mv:

(7) $$F_{impact}=\frac{mv}{t}=\frac{m\sqrt{2gh}}{t}$$

where t is the collision time which was set to 1 ms [34]. The substrate was fully attached and fixed to the specimen support to avoid change in collision time due to warping of the substrate. Additionally, because the ball collision surface and material were the same for both substrates, t was assumed to be the same.

**Figure 8:**
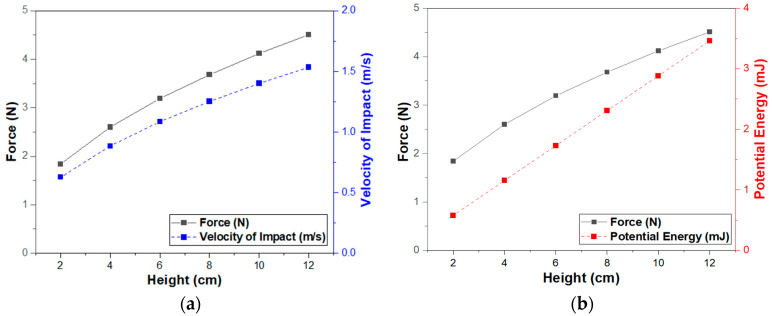
Theoretically calculated impact force ($F_{impact}$), velocity of impact ($v_{impact}$), and potential energy: (**a**) impact force and velocity of impact as a function of drop height; (**b**) impact force and potential energy as a function of drop height.

Figure 8 shows the variation of theoretically calculated impact force ($F_{impact}$), velocity of impact ($v_{impact}$), and potential energy as a function of the drop height for a 2.94 g ceramic ball. These analytical results clearly show that magnitudes of $F_{impact}$, $v_{impact}$, and potential energy increase with increasing drop height.

**Figure 9:**
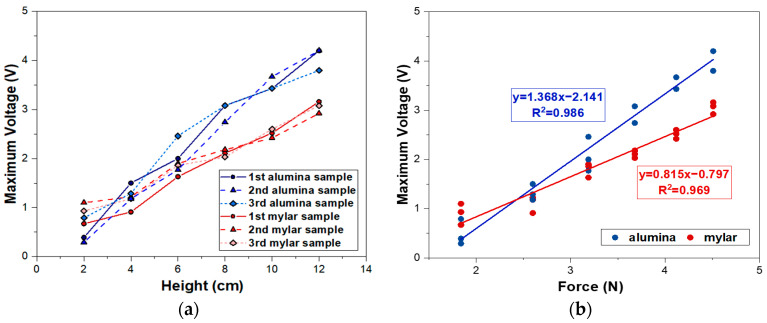
Comparison of impact force on the alumina and mylar substrates: (**a**) maximum voltage response as a function of height; (**b**) calibration curve of sensitivity of the sensor.

The maximum output voltage of the sensor resulting from the calculated impact force is shown in Figure 9. Both sensors’ maximum output voltage on the alumina and mylar substrates increased as the height of the ball drop increased, as shown in Figure 9a. For a drop height of 2 cm, 4 cm, 6 cm, 8 cm, 10 cm, and 12 cm, on average, the maximum output voltage of the sensor was 0.5 V, 1.3 V, 2.1 V, 4.0 V, 3.5 V, and 4.1 V for the alumina substrate and 0.9 V, 1.1 V, 1.8 V, 2.1 V, 2.5 V, and 3.1 V for the mylar substrate, respectively. The sensors on the alumina substrate provide a larger maximum output voltage than those on the mylar substrate, implying that the sensor’s responsivity to the impact force on the alumina substrate is higher than that on the mylar substrate. This could be attributed to a difference in substrate toughness, as polymers exhibit much higher toughness than ceramics [38]. It is deduced that the polymer-based mylar substrate absorbed more impact force than the ceramic-based alumina substrate. Through the maximum voltage and calculated impact force curve, the linearity of the sensor was observed on both substrates, which is required for real sensor applications due to the reduced complexity of signal processing. The sensitivity can be expressed in Equation (8) by correlating the output voltage with the applied force [39].

(8) $$Sensitivity=\frac{Output\;Voltage\;}{Applied\;Force}\;\left(V/N\right)$$

As shown in Figure 9b, a linear regression model was used to fit the data and calculate the sensitivity.

Sensitivities of 1.368 V/N and 0.815 V/N with correlation coefficients of 0.986 and 0.969 by the linear fit were obtained for sensors based on alumina and mylar substrates, respectively. The results indicate that our sensors’ sensitivity is higher than that of a previous piezoelectric sensor (0.0758 V/N) [36]. Moreover, the sensitivity of the sensor on the alumina substrate was approximately 70% higher than that on the mylar substrate, indicating that the sensitivity varied significantly depending on the substrate material. In other words, even if the same piezocomposite is used, the characteristics of a device could be altered depending on the substrate’s material properties. Research on various devices, including sensors, actuators, transducers, and energy harvesters, are focusing on the development of piezoelectric material itself [40,41]. However, in order to obtain the desired device’s characteristics, an appropriate substrate should be considered as a component of the device. In our case, alumina-based sensors are suitable for applications requiring high sensitivity such as displays and energy harvesters because of high sensitivity and rigidity. The flexibility of mylar-based sensors makes them appropriate for wearable devices.

### 3.4. Tactile Sensing

**Figure 10:**
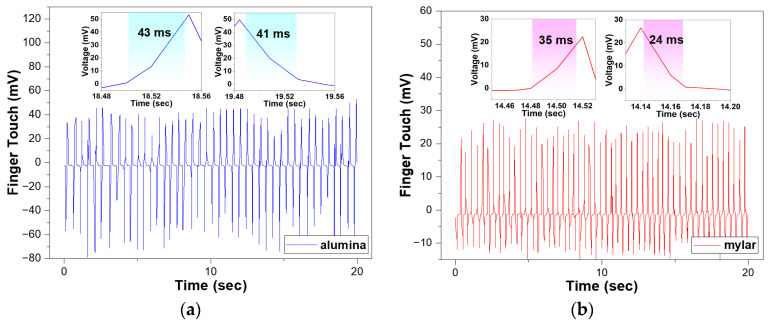
Output voltage and response time (inset: rising time and falling time) of the 0-3 PZT–PVB composite sensor to finger touch: (**a**) alumina substrate; (**b**) mylar substrate.

To verify the sensor’s capability as a tactile sensor, we measured the variation of output voltage when finger touch was repeatedly applied to the sensor. Figure 10 shows the output voltage response to repeated finger touch for 20 s on sensors based on the alumina and mylar substrates. The force of finger touch applied to the sensor was approximately 0.12 N, calculated by the integral of applied forced of the contact time. Applied force can be obtained by Equation (8) by substituting output voltage and sensitivity of the sensor. The sensor showed an immediate response to finger touch on both substrates. The peak voltage of the sensor was 28 mV on the mylar substrate and 53.3 mV on the alumina substrate, which was approximately 1.9 times higher than the sensor on the mylar substrate. The response behaviors are similar to the ball drop test, indicating that the sensor on the alumina substrate is more sensitive.

The response time, which means how fast the sensor changes its output signal in response to an external stimulus, can be obtained by applying periodic dynamic pressure to the sensor. It was determined by the time needed for the response voltage to increase or decrease from 10% to 90% of the output voltage. The rising time and falling time of the sensor were 35 ms and 24 ms on the mylar substrate and 43 ms and 41 ms on the alumina substrate. The results show that the response is faster than that of a previous piezoelectric sensor, which exhibited 87 ms and 64 ms [42]. The 0-3 PZT–PVB composite sensors on both the alumina and mylar substrates were confirmed to detect finger touch with a fast response, indicating that they are both suitable for use as tactile sensors.

## 4. Conclusions

In this study, tactile sensors made of 0-3 PZT–PVB composite on alumina and mylar substrates were fabricated using the screen-printing technique. The piezoelectric coefficients ($d_{33}$, $g_{33}$) of sensors printed on different substrates and poled at various temperatures were compared. Owing to mylar’s limited heat resistance, the maximum poling temperature was set at 150 °C. The ball drop test and finger touch test were performed in order to evaluate the sensor’s performance as a tactile sensor. The piezoelectric charge coefficient $d_{33}$ of the composites poled at 150 °C turned out to be 50% higher than that of the composites poled at 25 °C. The composites on the mylar substrate had $d_{33}$ and $g_{33}$ values that were 32.1% and 31.5% larger than those of the alumina substrate, respectively. This could be attributed to the bending effect of the substrate. The sensor on the alumina substrate achieved a sensitivity of 1.368 V/N, which was 1.68 times higher than on the mylar substrate. The rising time of the sensor was 43 ms on the alumina substrate and 35 ms on the mylar substrate. The 0-3 PZT–PVB composites were demonstrated to be a good candidate for the tactile sensor owing to their high sensitivity and fast response time. The 0-3 PZT–PVB composite tactile sensors can be used in a variety of applications, including energy harvesting, display, wearables, robotics, and electronic skin.

## Figures and Tables

**Table 1:** Rate of increase in the piezoelectric charge coefficient ($d_{33}$) according to the poling temperatures.

Temperature of Poling (°C)	$\mathrm{Increase}\;\mathrm{in}\;\mathrm{the}\;\mathrm{Piezoelectric}\;\mathrm{Charge}\;\mathrm{Coefficient}\;(d_{33})$ (%)	
	Alumina	Mylar
25 vs. 120	24.3	52.8
120 vs. 150	27.9	33.4
25 vs. 150	59.0	103.8
